# The impact of left ventricular geometry on left atrium phasic function in obstructive sleep apnea syndrome: a multimodal echocardiography investigation

**DOI:** 10.1186/s12872-021-02018-1

**Published:** 2021-04-24

**Authors:** Yong Zhang, Wen Shui, Yun Tian, Zhenxia Zhang, Juan Li, Jian Wang

**Affiliations:** 1 Medical imaging department of Shanxi Medical University; Department of Ultrasound, First Hospital of Shanxi Medical University, 85 Jiefang Nan Road, Taiyuan, 030001 Shanxi China; 2 Department of Respiratory, First Hospital of Shanxi Medical University, 85 Jiefang Nan Road, Taiyuan, 030001 Shanxi China

**Keywords:** Obstructive sleep apnea, Left ventricular geometry, Left atrium, Phasic function, Speckle-tracking echocardiography, Three-dimensional echocardiography

## Abstract

**Background:**

Left ventricular geometry and left atrium (LA) enlargement are risk factors for cardiovascular disease. However, reports on the relationship between left ventricular geometry and LA volume yielded contradictory findings, and LA phasic function remains unclear. Hence, this study aimed to investigate the influence of left ventricular geometry on LA volume and phasic function in patients with obstructive sleep apnea syndrome (OSAS) via a multimodal echocardiographic approach.

**Methods:**

In this cross-sectional study, 221 patients with OSAS (age 20–68 years, mean age 45.27 ± 12.50 years) underwent clinical evaluation, polysomnography, and multimodal echocardiographic examination with two-dimensional echocardiography (2DE), two-dimensional speckle-tracking echocardiography (2D-STE) and three-dimensional echocardiography (3DE). Based on conventional classification of left ventricular geometry, patients with OSAS were divided into four groups: normal geometry (NG), concentric remodeling (CR), concentric hypertrophy (CH), and eccentric hypertrophy (EH).

**Results:**

Based on 2DE and 3DE, the LA volumes and indices gradually increased from NG to CH. Additionally, 2DE and 3DE LA maximum volume index (LAVImax) were higher in patients with CH and EH than in patients with NG and CR (*P* < 0.05). The reservoir function, estimated by LA total emptying fraction (LA TotEF) was lower in patients with CH than in patients with NG in 2DE and 3DE (both, *P* < *0.05*). Also, LA conduit function, evaluated by LA passive emptying fraction (LA PassEF) was lower in patients with CH than in patients with NG and CR, and in patients with EH than in those with NG in 2DE and 3DE (all, *P* < *0.05*). The LA booster pump function, evaluated by LA active emptying fraction (LA ActEF) showed no statistically significant difference in 2DE; however, it was greater in patients with CH than in those with NG in 3DE. Similar results were obtained by 2D-STE, and CH was significantly associated with LA strain during systole (LAS-S, *β* = − 0.546, 95%CI: − 6.371–(− 3.444); *P* < *0.001*), early diastole (LAS-E, *β* = − 0.636, 95%CI: − 9.532–(− 5.710); *P* < *0.001*), and late diastole (LAS-A, *β* = − 0.450, 95%CI: 1.518–3.909; *P* < *0.001*) in multiple linear regression.

**Conclusions:**

The LA phasic function changed with left ventricular geometry via multimodal echocardiography. CH had the most notable negative effect on the maximum volume and phasic function of the LA.

**Supplementary Information:**

The online version contains supplementary material available at 10.1186/s12872-021-02018-1.

## Background

Obstructive sleep apnea syndrome (OSAS) is a prevalent sleep-related breathing disorder and an independent risk factor for cardiovascular disease [1]. Notably, abnormal left ventricular geometry with diastolic dysfunction and left atrium (LA) enlargement are common symptoms among patients with OSAS [2–4]. These have also been identified in patients without underlying cardiovascular disease [5]. Moreover, both conditions contribute to cardiovascular morbidity and mortality [6].

Multiple previous studies have revealed that left ventricular geometry is independently associated with LA enlargement. A study using the linear method of two-dimensional echocardiography reported that LA enlargement was related to the presence of eccentric hypertrophy [7]. Another study reported that LA enlargement, evaluated by LA volume through the area-length method of two-dimensional echocardiography, was significantly associated with concentric hypertrophy compared to eccentric hypertrophy [8]. Similarly, a study using the area-length method of two-dimensional echocardiography with larger sample sizes revealed that left atrium enlargement was associated with concentric hypertrophy and eccentric hypertrophy [9]. Furthermore, a previous study reported that LA enlargement estimated by diameter or volume was closely related to left ventricular mass index but was not related to left ventricular geometry [10].

Nevertheless, previous studies evaluated LA function using a single echocardiographic method and yielded contradictory findings. Additionally, existing echocardiographic techniques, including two-dimensional speckle tracking technique and three-dimensional echocardiography, are useful for evaluating LA size and phase function [11]. Therefore, our study was designed to investigate the influence of left ventricular geometry on LA enlargement and LA phasic function combined with various echocardiographic methods in patients with OSAS.

## Methods

### Study population

This cross-sectional study was approved by the Institutional Ethics Committee of Shanxi Medical University First Hospital. All patients were enrolled after providing written informed consent and all underwent physical examination, overnight polysomnography, blood biochemistry, and multimodal echocardiography.

**Fig. 1:**
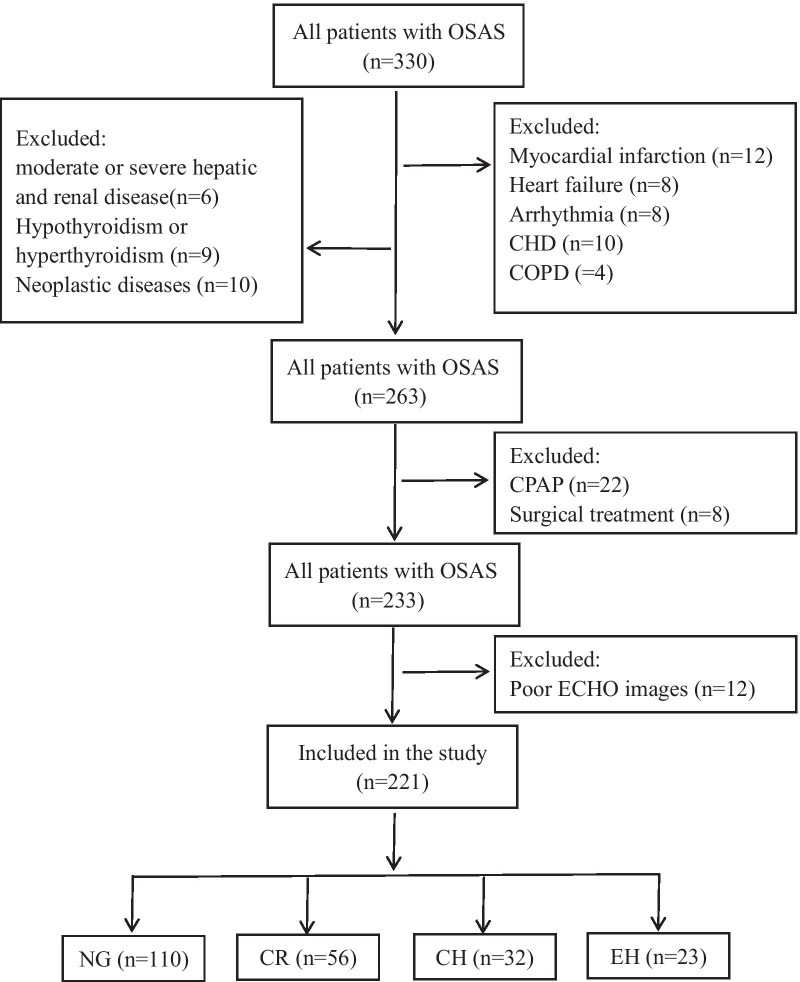
Study flowchart. OSAS: Obstructive sleep apnea syndrome; COPD: Chronic obstructive pulmonary disease; CPAP: continuous positive airway pressure; CHD: Coronary heart disease; ECHO: Echocardiography

Between January 2019 and December 2019, a total of 221 patients diagnosed with OSAS from Sleep Units in the First Hospital of Shanxi Medical University were enrolled (167 men, 54 women; age, 20–68 years) (Fig. 1).

The inclusion criteria were age > 18 years and patients with an apnea–hypopnea index (AHI) ≥ 5 events/hour. Briefly, AHI is the sum of the average number of apnea and hypopnea per hour [12]. Apnea is defined as a loss or significant decrease (≥ 90% from baseline) of oral and nasal airflow during sleep for ≥ 10 s. Hypopnea is defined as a 30% or more increase in oral-nasal airflow from baseline during sleep accompanied by a 3% or more decrease in blood oxygen saturation for a duration ≥ 10 s. The exclusion criteria included the following: myocardial infarction, heart failure, arrhythmia, coronary heart disease (CHD), moderate or severe valvular regurgitation or stenosis, left ventricular ejection fraction < 50% (modified Simpson method), moderate or severe hepatic and renal disease, chronic obstructive pulmonary disease (COPD), hypothyroidism or hyperthyroidism, and neoplastic disease. Moreover, patients who received continuous positive airway pressure (CPAP) or surgical treatment and had unsatisfactory echocardiographic images were excluded from the study.

Physical examination was performed in all patients. Blood pressure (systolic blood pressure [SBP] and diastolic blood pressure [DBP]) was measured using a mercury sphygmomanometer on the naked right arm with patients in a seated point position after 30 min of rest. Hypertension was defined as systolic blood pressure (BP) ≥ 140 mmHg or diastolic blood pressure ≥ 90 mmHg or being treated with hypertensive medication [13]. According to the 1999 WHO diagnostic criteria, diabetes was defined as FPG ≥ 7.0 mmol/L and/or blood glucose ≥ 11.1 mmol/L at 2 h postprandial or having been treated with diabetes medication. Obesity was defined as body mass index (BMI) ≥ 28 kg/m^2^, and dyslipidemia was defined as total cholesterol (TC) ≥ 5.17 mmol/L, triglycerides (TG) ≥ 1.7 mmol/L, high-density lipoprotein (HDL) < 1.03 mmol/L, and low-density lipoprotein (LDL) ≥ 3.33 mmol/L [14]. Body mass index (BMI) was calculated as the weight divided by height squared.

Blood samples were drawn from the peripheral vein the morning after PSG. Total cholesterol (TC), triglycerides (TG), high-density lipoprotein (HDL), low-density lipoprotein (LDL), and fasting blood glucose (Glu) levels were measured using an automatic biochemical analyzer (OLYMPUSAU5400, Japan).

### Polysomnography

All patients underwent overnight polysomnography in sleep units using standard recording techniques (SOMNO screen V series; German). Sleep recordings, including the apnea–hypopnea index (AHI), mean oxygen saturation (Mean-SaO_2_), lowest oxygen saturation (lowest SaO_2_), and percentage of total sleep time when blood oxygen saturation was less than 90% (T90), were obtained by a skilled technician based on the American Academic Sleep Medicine (AASM) 2017 criteria [15]. The AHI was defined as the number of apnea and hypopnea events per hour of sleep, and more than five were required for diagnosis.

### Echocardiography

Based on the recommendations of the American Society of Echocardiography [16], all patients underwent transthoracic echocardiographic examinations, including two-dimensional echocardiography (2DE), two-dimensional speckle-tracking echocardiography (2D-STE), and three-dimensional echocardiography (3DE), using EPIQ 7C Color Doppler Ultrasound (PHILIPS), with a S5-1and X5-1 probe and a frequency of 1.0–5.0 MHz. All patients were positioned in the left lateral decubitus position and connected to an electrocardiogram (ECG).

Left ventricular end-diastolic diameter (LVEDD), interventricular septum thickness (IVST), and left ventricular posterior wall thickness (LVPWT) were measured on the parasternal long-axis view. The early diastolic peak flow velocity (E) and late diastolic peak flow velocity (A) were measured using conventional pulsed Doppler imaging in the apical four-chamber view. Tissue Doppler imaging on the apical four-chamber view was used to measure mitral annular early diastolic myocardial velocity (e). Left ventricular ejection fraction was calculated using Simpson’s biplane method; meanwhile, left ventricular mass (LVM) was calculated while the left ventricular mass index (LVMI) was indexed to the body surface area by using the following formula [16]: 0.8 × 1.04 [ (LVEDD + IVST + PWT) ^3^ -LVEDD^3^]) + 0.6. The cutoff values of LVMI were 95 g/m^2^ for women and 115 g/m^2^ for men. Relative wall thickness (RWT), with a cutoff value of 0.42 [13], was calculated using the following formula: 2 × LVPWT/LVEDD.

Left ventricular geometric patterns were defined based on LVMI and RWT [16]: normal geometry (NG, normal LVMI and RWT), concentric remodeling (CR, normal LVMI, and increased RWT), concentric hypertrophy (CH, increased LVMI and RWT), and eccentric hypertrophy (EH, increased LVMI, and normal RWT).

### Two-dimension echocardiography (2DE) assessment of the left atrium volumes

The LA volumes (LAVs), including left atrial maximum volume (LAVmax), left atrium minimum volume (LAVmin), and left atrial pre-contraction volume (LAVpre), were measured using a biplane method in four-chamber and two-chamber views. The LAVmax was measured just before the mitral valve opening (the terminal of the T wave on the ECG), LAVmin was measured just after mitral valve closure (the peak of the R wave on the ECG), and LAVpre was measured at the onset of atrial systole (the peak of the P wave on the ECG). All LA volumes were indexed to body surface area (BSA).

### Two-dimensional speckle-tracking echocardiography (2D-STE) assessment of the left atrium strain

All dynamic images of the four-chamber and two-chamber views were obtained using conventional two-dimensional echocardiography for three consecutive cardiac cycles. All images were digitally stored, while offline images were analyzed using QLAB-Philips software (version 10; Philips Medical Systems).

The software automatically tracked the LA endocardium and manually adjusted the region of interest when speckle tracking was insufficient. The software then calculated six segments of the LA strain and its overall strain. Finally, the overall LA strain, including LA strain during systole (LAS-S) and LA strain during late diastole (LAS-A), combined the average of the values obtained for the four-chamber and two-chamber views. The LAS-S was measured when the aortic valve was closed, while LAS-A was measured at the beginning of the P wave of the ECG. The LA strain during early diastole (LAS-E) was defined as the difference between LAS-S and LAS-A [17].

### Three-dimensional echocardiography (3DE) assessment of the left atrium volumes

An X5-1 matrix-array transducer was used to acquire the “full-volume” for 4 consecutive cardiac cycles, and all images were digitally stored; meanwhile, they were analyzed offline using the QLAB-Philips software (version 10; Philips Medical Systems). The LA volumes were analyzed using the following anatomical landmarks: the septal, lateral, anterior, and posterior points on the atrial surface of the mitral annulus and the higher point of the LA roof. The LA appendages and pulmonary veins were excluded. Subsequently, the software automatically tracked the LA endocardium and manually adjusted the region of interest when necessary. Finally, the volume-time curve of the LA was obtained [18]. Measurement of LAVs was similar to measurement of the two-dimensional echocardiography as described above.

Based on the above LAVs, the parameters of LA function were calculated as follows [19]: LA total emptying volume (LA TotEV): LAVmax- LAVmax; LA passive emptying volume (LA PassEV): LAVmax- LAVpre; LA active emptying volume (LA ActEV): LAVpre-LAVmin; LA total emptying fraction (LA TotEF): LA TotEV/LAVmax × 100; LA passive emptying fraction (LA PassEF): LA PassEV/LAVmax × 100; LA active emptying fraction (LA ActEF): LA ActEV/LAVpre × 100.

### Statistical analysis

Continuous variables were evaluated for normal distribution using the one-way Kolmogorov–Smirnov test. Normally distributed continuous variables were presented as mean ± standard deviation and analyzed using one-way ANOVA with Bonferroni post hoc test. Categorical variables were presented as percentages and were analyzed using the chi-square test. Multiple linear regression analysis was used to establish the relationship between left ventricular geometry and LAVImax, as well as left ventricular phasic function after adjusting for age, gender, BMI, SBP, diabetes mellitus, dyslipidemia, AHI, and LAMI. A *P*-value < 0.05 (*P* < 0.05) was considered statistically significant. All data were analyzed using SPSS 22.0 for Windows (IBM, Armonk, NY, USA). The figures were generated using GraphPad Prism 8.

## Results

### Clinical, polysomnographic, and blood biochemical parameters in the four left ventricular geometric patterns

As shown in Table 1, we recruited 221 patients with OSAS, 110 patients with normal geometry, 56 patients with concentric remodeling, 32 patients with concentric hypertrophy, and 23 patients with eccentric hypertrophy. In contrast with normal geometry and concentric remodeling, patients with concentric hypertrophy and eccentric hypertrophy were associated with older age (*P* < 0.05). Patients with concentric hypertrophy and eccentric hypertrophy had higher values of SBP, DBP, hypertension, AHI, and T90, but had lower values of mean-SaO_2_ than those with normal geometry (*P* < 0.05). Regarding sex, BMI, obesity, diabetes mellitus, dyslipidemia, medications (including ARBs/ACEIs, Beta-blockers, CCB, diuretics, statin and antidiabetic), heart rate, lowest- SaO_2,_ and blood biochemical values, no statistical difference was found among the four groups (Table 1).

**Table 1:** Clinical, polysomnographic and blood biochemical parameters in the four left ventricular geometric patterns

	NG (n = 110)	CR (n = 56)	CH (n = 32)	EH (n = 23)	*P*
Age (years)	41.80 ± 1.22	44.27 ± 1.45	51.88 ± 1.73^ab^	55.09 ± 2.03^ab^	< 0.001
Female (%)	24.5	23.2	25	26.1	0.994
BMI (kg/m^2^)	27.75 ± 0.39	28.38 ± 0.53	28.08 ± 0.26	28.37 ± 0.30	0.703
SBP (mmHg)	129.36 ± 1.32	132.43 ± 1.76	138.50 ± 1.73^a^	138.17 ± 2.37^a^	0.001
DBP (mmHg)	77.53 ± 1.10	79.30 ± 1.44	86.44 ± 1.87^a^	84.30 ± 1.69^a^	< 0.001
HR (bpm)	64.91 ± 0.59	66.82 ± 0.93	65.37 ± 1.29	65.87 ± 1.66	0.389
AHI (events/h)	36.86 ± 2.49	46.15 ± 3.60	54.18 ± 5.31^a^	51.83 ± 4.86^a^	0.003
Mean-SaO_2_ (%)	92.54 ± 2.88	91.41 ± 3.52	90.89 ± 3.78^a^	90.96 ± 2.93^a^	0.014
Lowest-SaO_2_ (%)	74.95 ± 1.33	71.73 ± 2.06	69.69 ± 2.67	71.52 ± 3.58	0.251
T90 (%)	13.51 ± 1.36	19.17 ± 2.97	27.48 ± 3.43^a^	24.05 ± 4.34^a^	< 0.001
TC (mmol/L)	4.54 ± 0.06	4.44 ± 0.09	4.65 ± 0.17	4.63 ± 0.15	0.561
TG (mmol/L)	2.01 ± 0.09	1.92 ± 0.10	2.15 ± 0.13	1.95 ± 0.15	0.673
HDL (mmol/L)	1.17 ± 0.03	1.13 ± 0.04	1.16 ± 0.03	1.09 ± 0.04	0.493
LDL (mmol/L)	2.60 ± 0.07	2.32 ± 0.11	2.61 ± 0.16	2.72 ± 0.22	0.111
Glu (mmol/L)	5.26 ± 0.07	5.24 ± 0.14	5.56 ± 0.19	5.38 ± 0.22	0.401

NG: normal geometry; CR: concentric remodeling; CH: concentric hypertrophy; EH: eccentric hypertrophy; BMI: body mass index; SBP: systolic blood pressure; DBP: diastolic blood pressure; AHI: apnea hypopnea index; Mean-SaO2: mean oxygen saturation; Lowest-SaO2: lowest oxygen saturation; T90: percentage of total sleep time when blood oxygen saturation is less than 90%; TC: Total cholesterol; TC: triglyceride; HDL: High-density lipoprotein; LDL: Low-density lipoprotein; Glu: Glucose

^a^*p* < 0.05 for normal geometry; ^b^*p* < 0.05 for concentric remodeling

### Echocardiographic characteristics of the left ventricle in the four left ventricular geometric patterns

As shown in Table 2, patients with concentric hypertrophy and eccentric hypertrophy demonstrated a significantly larger LA dimension than those with normal geometry and concentric remodeling (*P* < 0.05). Similarly, both had greater interventricular septal thickness, left ventricular posterior wall thickness, and left ventricular mass index (*P* < 0.05). Furthermore, patients with concentric remodeling and concentric hypertrophy exhibited higher relative wall thickness than those with normal geometry and eccentric hypertrophy (*P* < 0.05). The E/A ratio gradually decreased, while the E/e ratio gradually increased from normal geometry to concentric hypertrophy. Regarding LVEF, no statistical difference was found among the four groups (Table 2).

**Table 2:** Echocardiographic Characteristics of left ventricular in the four left ventricular geometric patterns

	NG (n = 110)	CR (n = 56)	CH (n = 32)	EH (n = 23)	*P*
LAD (cm)	3.25 ± 0.33	3.40 ± 0.31^a^	3.74 ± 0.14^ab^	3.79 ± 0.14^ab^	< 0.001
LVEDD (cm)	4.72 ± 0.03	4.61 ± 0.05	4.99 ± 0.08^ab^	5.32 ± 0.07^abc^	< 0.001
IVST (cm)	0.92 ± 0.08	0.98 ± 0.14^a^	1.16 ± 0.08^ab^	1.09 ± 0.12^abc^	< 0.001
LVPWT (cm)	0.87 ± 0.08	1.00 ± 0.12^a^	1.13 ± 0.09^ab^	1.02 ± 0.17^ac^	< 0.001
LVMI (g/m^2^)	75.44 ± 1.25	82.85 ± 2.12^a^	117.73 ± 2.13^ab^	115.69 ± 2.14^ab^	< 0.001
RWT	0.37 ± 0.02	0.44 ± 0.03^a^	0.45 ± 0.07^ab^	0.39 ± 0.05^abc^	< 0.001
E/A	0.97 ± 0.03	0.93 ± 0.04	0.79 ± 0.03^ab^	0.87 ± 0.07^ac^	0.018
E/e	6.54 ± 0.25	7.72 ± 0.29^a^	9.77 ± 0.41^ab^	8.87 ± 0.68^a^	< 0.001
LVEF (%)	65.99 ± 0.52	66.31 ± 0.90	66.34 ± 0.93	63.71 ± 1.73	0.343

LAD: left atrium dimension; LVEDD: left ventricular end-diastolic diameter; IVST: inter-ventricular septum thickness; LVPWT: left ventricular posterior wall thickness; LVMI: left ventricular mass index; RWT: relative wall thickness; LVEF: left ventricular ejection fraction

^a^*p* < 0.05 for normal geometry; ^b^*p* < 0.05 for concentric remodeling; ^c^*p* < 0.05 for concentric hypertrophy

### Left atrium volumes and strains in the four left ventricular geometric patterns

**Fig. 2:**
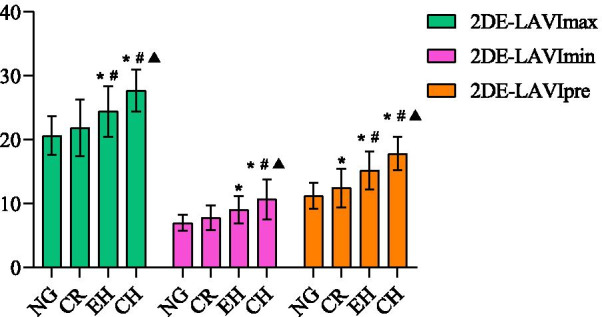
The comparison of 2DE-left atrium volume indices (LAVImax, LAVImin, LAVIpre) among the different left ventricular geometric patterns. NG: Normal geometry; CR: Concentric remodeling; EH: Eccentric hypertrophy; CH: Concentric hypertrophy; *P < 0.05 versus NG; ^#^*P* < 0.05 versus CR; ^▲^*P* < 0.05 versus EH

**Fig. 3:**
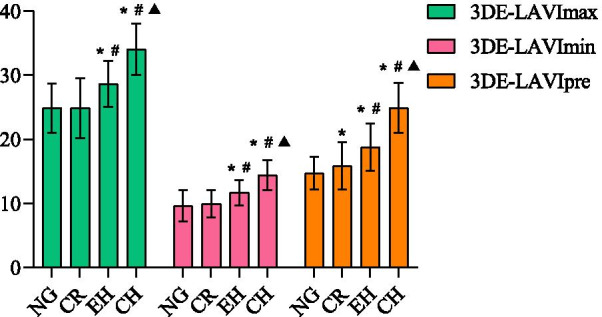
The comparison of 3DE-left atrium volume indices (LAVImax, LAVImin, LAVIpre) among the different left ventricular geometric patterns. NG: Normal geometry; CR: Concetric remodeling; EH: Eccentric hypertrophy; CH: Concentric hypertrophy; *P < 0.05 versus NG; ^#^*P* < 0.05 versus CR; ^▲^*P* < 0.05 versus EH

As demonstrated in Table 3, 2DE- and 3DE LA volumes and indices (maximum, minimum, pre-contraction) gradually increased from normal geometry to concentric hypertrophy. The 3DE-LAVmax showed a statistically significant difference among the four groups, while 2DE-LAVmax was statistically significant among all four groups, except for concentric hypertrophy and eccentric hypertrophy. In addition, 2DE- and 3DE-LAVImax revealed a statistically significant difference among the other left ventricular geometries, except for normal geometry and concentric remodeling (Figs. 2 and 3). Patients with concentric hypertrophy had higher 3DE-LAVmin and 3DE-LAVImin than those with normal geometry, concentric remodeling, and eccentric hypertrophy, as well as higher 2DE- LAVmin and 2DE-LAVImin than those with normal geometry and concentric remodeling (*P* < 0.05) (Figs. 2 and 3). Additionally, 2DE-and 3DE-LAVpre and LAVIpre were significantly different in patients with left ventricular geometry (*P* < 0.05) (Figs. 2 and 3).

**Table 3:** Left atrium volumes and strains in the four left ventricular geometric patterns

	NG (n = 110)	CR (n = 56)	CH (n = 32)	EH (n = 23)	*P*
*2DE LAV variables*					
LAVmax (ml)	39.14 ± 5.40	41.81 ± 8.04^a^	49.26 ± 4.47^ab^	45.90 ± 5.56^ab^	< 0.001
LAVImax (ml/m^2^)	20.66 ± 3.05	21.88 ± 4.45	27.66 ± 3.29^ab^	24.42 ± 4.01^abc^	< 0.001
LAVmin (ml)	13.25 ± 2.40	14.90 ± 3.43^a^	19.04 ± 5.13^ab^	16.95 ± 3.40^a^	< 0.001
LAVImin (ml/m^2^)	6.99 ± 1.27	7.80 ± 1.90	10.71 ± 3.12^ab^	9.04 ± 2.17^ac^	< 0.001
LAVpre (ml)	21.36 ± 4.03	23.72 ± 5.74^a^	31.75 ± 4.02^ab^	28.51 ± 4.04^ab^	< 0.001
LAVIpre (ml/m^2^)	11.25 ± 2.04	12.40 ± 3.01^a^	17.82 ± 2.59^ab^	15.20 ± 2.94^abc^	< 0.001
LA TotEV (ml)	25.90 ± 3.86	26.91 ± 5.42	30.22 ± 4.63^ab^	28.95 ± 3.10^a^	0.005
LA PassEV (ml)	17.79 ± 2.60	18.09 ± 3.12	17.51 ± 1.92	17.39 ± 2.37	0.655
LA ActEV (ml)	8.11 ± 2.61	8.82 ± 3.30	12.71 ± 3.88^ab^	11.56 ± 1.83^ab^	< 0.001
*3DE LAV variables*					
LAVmax (ml)	46.85 ± 4.80	49.00 ± 5.47^a^	60.60 ± 4.66^ab^	53.77 ± 3.78^abc^	< 0.001
LAVImax (ml/m^2^)	24.86 ± 3.85	25.84 ± 4.68	34.05 ± 3.98^ab^	28.60 ± 3.59^abc^	< 0.001
LAVmin (ml)	18.04 ± 3.83	18.89 ± 2.95	25.57 ± 3.31^ab^	21.95 ± 2.74^abc^	< 0.001
LAVImin (ml/m^2^)	9.60 ± 2.43	9.96 ± 2.13	14.37 ± 2.23^ab^	11.69 ± 1.96^abc^	< 0.001
LAVpre (ml)	27.78 ± 3.71	30.11 ± 5.51^a^	44.36 ± 5.76^ab^	35.25 ± 5.54^abc^	< 0.001
LAVIpre (ml/m^2^)	14.72 ± 2.49	15.87 ± 3.66	24.93 ± 3.91^ab^	18.78 ± 3.68^abc^	0.018
LA TotEV (ml)	28.80 ± 4.63	30.11 ± 3.84	35.03 ± 4.19^ab^	31.82 ± 1.66^ac^	< 0.001
LA PassEV (ml)	19.06 ± 4.93	18.89 ± 3.82	16.24 ± 2.97^ab^	18.53 ± 2.89	0.011
LA ActEV (ml)	9.74 ± 4.15	11.21 ± 4.43	18.79 ± 4.53^ab^	13.29 ± 3.88^ac^	< 0.001

LAVmax: left atrium maximum volume; LAVImax: left atrium maximum volume index; LAVmin: left atrium minimum volume; LAVImin: left atrium minimum volume index; LAVpre: left atrium pre-contraction volume; LAVIpre: left atrium pre-contraction volume index; LA TotEV: left atrium total emptying volume; LA PassEV: left atrium passive emptying volume; LA ActEV: left atrium active emptying volume

^a^*p* < 0.05 for normal geometry; ^b^*p* < 0.05 for concentric remodeling; ^c^*p* < 0.05 for concentric hypertrophy

Notably, 2DE-LA TotEV was greater in patients with concentric hypertrophy than in those with normal geometry and concentric remodeling; 3DE-LA TotEV was greater in patients with concentric hypertrophy than in those with normal geometry, concentric remodeling, and eccentric hypertrophy; 2DE- and 3DE-LA TotEV were greater in eccentric hypertrophy than in normal geometry. The 3DE-LA PassEV was lower in patients with concentric hypertrophy than in those with normal geometry and concentric remodeling. However, 3DE-LA PassEV showed no differences among the left ventricular geometric patterns. Patients with concentric hypertrophy and eccentric hypertrophy demonstrated a significantly larger 2DE-LA ActEV compared to those with normal geometry and concentric remodeling. Furthermore, patients with concentric hypertrophy revealed a significantly larger 3DE-LA ActEV compared to any of the left ventricular geometric patterns, and larger eccentric hypertrophy compared to those with normal geometry.

**Fig. 4:**
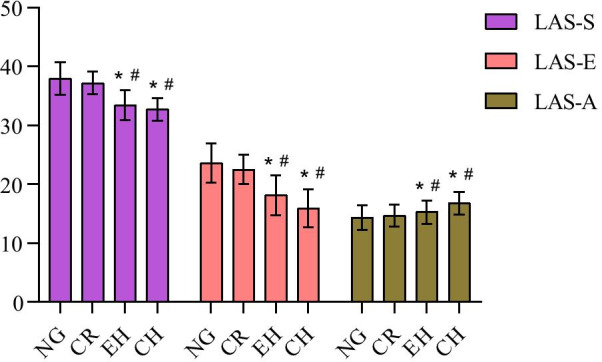
The comparison of left atrium strain parameters (LAS-S, LAS-E, LAS-A) among the different left ventricular geometric patterns. NG: Normal geometry; CR: Concetric remodeling; EH: Eccentric hypertrophy; CH: Concentric hypertrophy; *P < 0.05 versus NG; ^#^*P* < 0.05 versus CR; ^▲^*P* < 0.05 versus EH

The trend of change in left ventricular strains was similar to the change observed in LA volumes. The LAS-S and LAS-E values progressively decreased from normal geometry to concentric hypertrophy; however, LAS-A gradually increased. Patients with concentric hypertrophy and eccentric hypertrophy had higher LAS-S and LAS-E than those with normal geometry and concentric remodeling. The LAS-A was higher among patients with concentric hypertrophy than in those presenting any left ventricular geometric pattern (*P* < 0.05) (Fig. 4).

### Left atrium phasic function in the four left ventricular geometric patterns

**Fig. 5:**
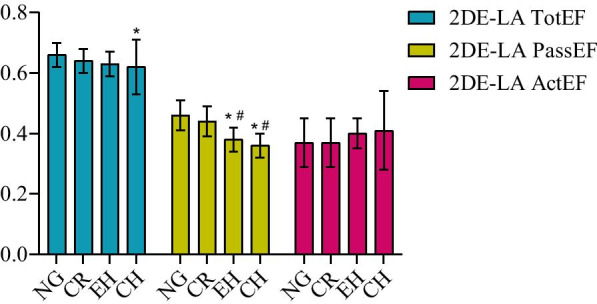
The comparison of 2DE-left atrium emptying fraction (TotEF, PassEF, ActEF) among the different left ventricular geometric patterns. NG: Normal geometry; CR: Concetric remodeling; EH: Eccentric hypertrophy; CH: Concentric hypertrophy; *P < 0.05 versus NG; ^#^*P* < 0.05 versus CR

**Fig. 6:**
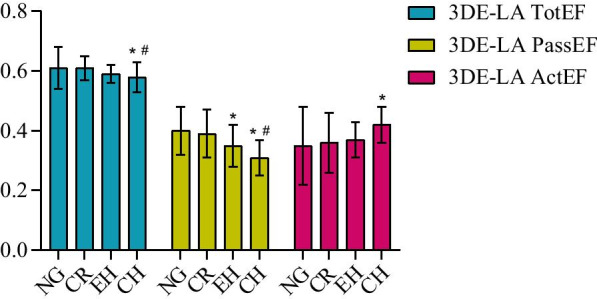
The comparison of 3DE- left atrium emptying fraction (TotEF, PassEF, ActEF) among the different left ventricular geometric patterns. NG: Normal geometry; CR: Concetric remodeling; EH: Eccentric hypertrophy; CH: Concentric hypertrophy; *P < 0.05 versus NG; ^#^*P* < 0.05 versus CR

As shown in Table 4, the LA reservoir, estimated by LA total emptying fraction (LA TotEF) and conduit function, estimated by LA passive emptying fraction (LA PassEF), gradually decreased from normal geometry to concentric hypertrophy. Nevertheless, LA booster function, estimated by LA active emptying fraction (LA ActEF), gradually increased in a similar direction. Patients with concentric hypertrophy had lower 2DE-LA TotEF compared to those with normal geometry and lower 3DE-LA TotEF compared to those with normal geometry and concentric remodeling (Figs. 5 and 6). Patients with concentric hypertrophy and eccentric hypertrophy had lower 2DE-LA PassEF compared to those with normal geometry and concentric remodeling. In addition, patients with concentric hypertrophy had lower 3DE-LA PassEF compared to those with normal geometry and concentric remodeling, and lower 3DE-LA PassEF in patients with eccentric hypertrophy compared to those with normal geometry (Figs. 5 and 6). Patients with concentric hypertrophy had higher 3DE-LA ActEF values than those with normal geometry. However, 2DE-LA ActEF showed no difference among the left ventricular geometric patterns (Figs. 5 and 6).

**Table 4:** Left atrium phasic function in the four left ventricular geometric patterns

	NG (n = 110)	CR (n = 56)	CH (n = 32)	EH (n = 23)	*P*
*Reservoir function*					
2DE-LA TotEF (%)	0.66 ± 0.04	0.64 ± 0.04	0.62 ± 0.09^a^	0.63 ± 0.04	< 0.001
3DE-LA TotEF (%)	0.61 ± 0.07	0.61 ± 0.04	0.58 ± 0.05^ab^	0.59 ± 0.03	0.009
2DE-LAS-S (%)	37.97 ± 2.74	37.20 ± 1.89	32.70 ± 1.93^ab^	33.44 ± 2.53^ab^	< 0.001
*Conduit function*					
2DE-LA PassEF (%)	0.46 ± 0.05	0.44 ± 0.05	0.36 ± 0.04^ab^	0.38 ± 0.04^ab^	< 0.001
3DE-LA PassEF (%)	0.40 ± 0.08	0.39 ± 0.08	0.31 ± 0.06^ab^	0.35 ± 0.07^a^	< 0.001
2DE-LAS-E (%)	23.62 ± 3.36	22.52 ± 2.48	15.95 ± 3.16^ab^	18.13 ± 3.37^ab^	< 0.001
*Pump function*					
2DE-LA ActEF (%)	0.37 ± 0.08	0.37 ± 0.08	0.41 ± 0.13	0.40 ± 0.05	0.098
3DE-LA ActEF (%)	0.35 ± 0.13	0.36 ± 0.10	0.42 ± 0.06^a^	0.37 ± 0.06	0.011
2DE-LAS-A (%)	14.35 ± 2.06	14.68 ± 1.85	16.75 ± 1.89^ab^	15.31 ± 1.98^c^	< 0.001

LA TotEF: left atrium total emptying fraction; LA PassEF: left atrium passive emptying fraction; LA ActEF: left atrium active emptying fraction; LAS-S: left atrium strain during systole; LAS-E: left atrium strain during early diastole; LAS-A: left atrium strain during late diastole

^a^*p* < 0.05 for normal geometry; ^b^*p* < 0.05 for concentric remodeling; ^c^*p* < 0.05 for concentric hypertrophy

### Multiple linear regression analysis of the relationship between the left ventricular geometry and LAVImax and LA phasic function

In order to clarify the independent influence of the left ventricular geometric patterns on LAVImax and LA phasic function, we performed multiple linear regression analyses, in which age, gender, BMI, SBP, diabetes mellitus, dyslipidemia, AHI, and LVMI were included in the model. As shown in Table 5, concentric hypertrophy was significantly associated with 2DE-LAVImax (*β* = 0.449, 95%CI: 3.592–7.436; *P* < *0.001*), while concentric and eccentric hypertrophy were associated with 3DE-LAVImax (*β* = 0.539, 95%CI: 5.946–9.761; *P* < *0.001* and *β* = 0.137, 95%CI: 0.290–4.306; *P* = *0.025*, respectively). Subsequently, concentric hypertrophy was associated with 2DE- and 3DE-LA TotEF (*β* = -0.219, 95%CI: -0.074- (-0.001); *P* = *0.046* and *β* = -0.276, 95%CI: -0.073- (-0.010); *P* = *0.010*, respectively). Moreover, concentric hypertrophy and eccentric hypertrophy were associated with 2DE-LA PassEF (*β* = -0.422, 95%CI: -0.148- (-0.052); *P* < *0.001* and *β* = -0.221, 95%CI: -0.111- (-0.010); *P* = *0.019*, respectively), while concentric hypertrophy, eccentric hypertrophy, and concentric remodeling were associated with 3DE-LA PassEF (*β* = -0.589, 95%CI: -0.126- (-0.070); *P* < *0.001*, *β* = -0.382, 95%CI: -0.103- (-0.044); *P* < *0.001*, and *β* = -0.138, 95%CI: -0.034- (-0.003); *P* = *0.018*, respectively). Only concentric hypertrophy was associated with 3DE-LA ActEF (*β* = 0.239, 95%CI: 0.005–0.144; *P* = *0.035*). Furthermore, concentric and eccentric hypertrophy were associated with 3DE-LAS-S, 3DE-LAS-E, and 3DE-LAS-A (all, *P* < *0.05*).

**Table 5:** Multiple linear regression analysis the relationship between left ventricular geometry and LAVI and LA phasic function (Adjusted age, sex, BMI, SBP, diabetes mellitus, dyslipidemia, AHI, LVMI)

	NG	CR				CH				EH			
		Beta	t	95%CI	*P*	Beta	t	95%CI	*P*	Beta	t	95%CI	*P*
2DE-LAVImax	Referent	0.097	1.796	− 0.094–2.022	0.074	0.449	5.656	3.592–7.436	< 0.001	0.137	1.892	− 0.082–3.964	0.060
3DE-LAVImax	Referent	0.076	1.681	− 0.154–1.946	0.076	0.539	8.116	5.946–9.761	< 0.001	0.137	2.256	0.290–4.306	0.025
*Reservoir function*													
2DE-LA TotEF (%)	Referent	− 0.007	− 0.095	− 0.021–0.019	0.925	− 0.219	− 2.021	− 0.074–(− 0.001)	0.046	− 0.117	− 1.172	− 0.062–0.016	0.243
3DE-LA TotEF (%)	Referent	− 0.134	− 1.857	− 0.034–0.001	0.065	− 0.276	− 2.601	− 0.073–(− 0.010)	0.010	− 0.134	− 1.381	− 0.056–0.010	0.169
2DE-LAS-S (%)	Referent	− 0.095	− 1.695	− 1.498–0.113	0.092	− 0.546	− 6.610	− 6.371–(− 3.444)	< 0.001	− 0.403	− 5.339	− 5.713–(− 2.632)	< 0.001
*Conduit function*													
2DE-LA PassEF (%)	Referent	− 0.088	− 1.269	− 0.043–0.009	0.206	− 0.422	− 4.134	− 0.148–(− 0.052)	< 0.001	− 0.221	− 2.370	− 0.111–(− 0.010)	0.019
3DE-LA PassEF (%)	Referent	− 0.138	− 2.381	− 0.034–(− 0.003)	0.018	− 0.589	− 6.888	− 0.126–(− 0.070)	< 0.001	− 0.382	− 4.897	− 0.103–(− 0.044)	< 0.001
2DE-LAS-E (%)	Referent	− 0.113	− 2.045	− 2.143–(− 0.039)	0.042	− 0.636	− 7.863	− 9.532–(− 5.710)	< 0.001	− 0.400	− 5.413	− 7.532–(− 3.512)	< 0.001
*Pump function*													
2DE-LA ActEF (%)	Referent	− 0.039	− 0.522	− 0.036–0.021	0.602	0.161	1.466	− 0.013–0.090	0.144	0.151	1.500	− 0.013–0.096	0.135
3DE-LA ActEF (%)	Referent	0.038	0.490	− 0.029–0.048	0.624	0.239	2.119	0.005–0.144	0.035	0.066	0.643	− 0.049–0.096	0.521
2DE-LAS-A (%)	Referent	0.082	1.193	− 0.260–1.056	0.234	0.450	4.476	1.518–3.909	< 0.001	0.194	2.117	0.093–2.609	0.035

LAVImax: left atrium maximum volume index; LA TotEF: left atrium total emptying fraction; LA PassEF: left atrium passive emptying fraction; LA ActEF: left atrium active emptying fraction; LAS-S: left atrium strain during systole; LAS-E: left atrium strain during early diastole; LAS-A: left atrium strain during late diastole

## Discussion

To our knowledge, this is the first study to evaluate the influence of left ventricular geometry on LA volume and phase function in patients with OSAS combined with multiple echocardiographic methods. Our main findings include the following: (1) LA volumes and indices showed an increasing pattern from normal geometry to concentric hypertrophy, and 2DE-LAVImax was associated with concentric hypertrophy, while 3DE-LAVImax was associated with concentric hypertrophy and eccentric hypertrophy; (2) LA reservoir and conduit function gradually increased from normal geometry to concentric hypertrophy, while LA booster pump function gradually decreased in a similar direction; (3) multiple linear regression analysis revealed that concentric hypertrophy was significantly associated with LA phasic function, and 3DE parameters were significantly associated with left ventricular geometric patterns compared to 2DE parameters.

An enlarged LA was associated with increased severity of OSAS and was evaluated by LA volume using 2DE and 3DE. Nevertheless, changes in LA volume remain unclear in patients with OSAS based on left ventricular geometry. Our findings showed that LA volumes and indices gradually increased from normal geometry to concentric hypertrophy. This may have been due to left ventricular overload and impaired myocardial relaxation caused by nocturnal apnea–hypopnea events, which may have triggered left ventricular diastolic dysfunction [20, 21]. Moreover, left ventricular diastolic function gradually decreased from normal geometry to concentric hypertrophy, resulting in increased left ventricular filling pressure and blocked LA blood flow into the left ventricle, and may explain the change in LA volume. Importantly, LAVImax played a vital role in predicting cardiovascular adverse events and was included in the guidelines [16]. However, previous findings on the relationship between LAVImax and left ventricular geometry are inconsistent. The results varied in that LAVImax was only related to eccentric hypertrophy, concentric hypertrophy, or both, and not related to left ventricular geometry [7–10]. The varying results among different studies may be due to differences in duration and degree of hypertension and the state of treatment.

Furthermore, the present study demonstrated that only concentric hypertrophy was associated with 2DE-LAVImax. This study was the same as the previous study, in which individual left ventricular geometric patterns were related to LAVImax; meanwhile, differences in our study may be due to differences in type of disease, blood pressure, and age. Importantly, we evaluated LAVImax using 3DE, suggesting that concentric and eccentric hypertrophy were associated with 3DE-LAVImax. This was due to the fact that 3DE was more precise than 2DE in the measurement of left ventricular volume and highly valuable in predicting the incidence of cardiovascular adverse events [16]. Our results confirm that the incidence of cardiovascular events and clinical prognosis differ in concentric and eccentric hypertrophy [22].

Previous studies evaluated the phasic function of LA using 3DE and 2D-STE, respectively. Consequently, they demonstrated that OSAS induces LA remodeling and dysfunction [23, 24]. Nevertheless, our study further revealed that LA phasic function was based on left ventricular geometry.

In addition, we found that LA reservoir function, estimated by LA TotEF and LAS-S, gradually decreased from normal geometry to concentric hypertrophy. This result may have been due to the fact that the hypertrophic wall of the left ventricle increased compression of the coronary artery and oxygen consumption of hypertrophic cardiomyocytes, causing hypoxia damage and sensitivity to ischemia of cardiomyocytes, further inducing fibrosis and decreased compliance in the left atrium [25]. Notably, this phenomenon is apparent with a larger LVMI. Furthermore, LVMI increased from a normal geometry to concentric hypertrophy. In contrast, LA TotEF decreased, and the LAS-S showed similar results. Multiple linear regression analysis revealed that concentric hypertrophy was significantly associated with LA reservoir function.

This investigation also demonstrated that LA conduit function, evaluated by LA PassEF and LAS-E, gradually decreased from normal geometry to concentric hypertrophy. Despite not being statistically significant, Oliveira et al. previously showed that 3DE-LA PassEF decreased in patients with OSAS compared to the control group [23]. Altekin et al. assessed LA phasic function using 2D-STE and reported that LAS-E gradually decreased from control to severe OSAS and was associated with AHI [24]. Similarly, Tadic and colleagues revealed similar findings that the LA conduit function decreased in patients with hypertension [26]. Here, SBP, DBP, and AHI gradually increased from normal geometry to concentric hypertrophy. Our results are consistent with those of previous studies. Multiple linear regression showed that concentric and eccentric hypertrophy were associated with 2DE- and 3DE-LA PassEF, and LA strain confirmed these results.

The LA booster pump function, evaluated by LA ActEF and LAS-A, gradually increased from normal geometry to concentric hypertrophy. Previous studies have shown that OSAS causes an increase in LA booster pump function [23, 24]. In addition, using velocity vector imaging, Yang et al. found that patients with hypertension and left ventricular hypertrophy had reduced LA conduit function and enhanced booster pump function [27]. Our results are in line with those of a previous study, possibly due to the compensatory increase in LA booster pump function, which is essential to maintain normal left ventricular diastolic filling with a decrease in LA reservoir and conduit function. Multiple linear regression revealed that concentric hypertrophy was associated with 3DE-LA ActEF; meanwhile, it was not associated with 2DE-LA ActEF. The LA strain validated the 3DE results, showing that concentric hypertrophy was associated with LAS-A. This was primarily due to the fact that the LA strain was more useful for the prediction of adverse cardiovascular events than the 2DE LA volume and more sensitive to these events. Furthermore, this could explain why patients with left ventricular hypertrophy and higher LVMI have increased LA booster pump function than those without left ventricular hypertrophy.

Our study has the following limitations. Due to its cross-sectional nature, we could not ascertain a direct causal relationship between left ventricular geometry and LA phase function; hence, further longitudinal studies are necessary. Also, the study could be carried out using the new classifications proposed by Dallas et al. [28] due to the relatively small sample size. Changes in LA phase function need to be further investigated in the future using a new classification system.

## Conclusions

Our findings suggest that LA phasic function changes with left ventricular geometry. Moreover, the reservoir and conduit function of the LA gradually decreased from normal geometry to concentric hypertrophy; nevertheless, the function of the booster pump gradually increased. Furthermore, concentric hypertrophy had the most significant negative impact on LAVImax and LA phasic function.

## Supplementary Information

**Additional file 1.** Ethical approval.

**Additional file 2.** Certificate of english editing.

## Data Availability

The datasets analyzed during the current study are available from the corresponding author upon request.

## Abbreviations

OSAS: Obstructive sleep apnea syndrome
AHI: Apnea–hypopnea index
SaO_2_: Oxygen saturation
2DE: Two-dimensional echocardiography
3DE: Three-dimensional echocardiography
2D-STE: Two-dimensional speckle-tracking echocardiography
LAVImax: Left ventricular maximum volume index
LAVImin: Left ventricular minimum volume index
LAVIpre: Left ventricular pre-contraction volume index
LA TotEF: Left ventricular total emptying fraction
LA PassEF: Left ventricular passive emptying fraction
LA ActEF: Left ventricular active emptying fraction
LV: Left ventricle
