# Supplementary material for: The impact of left ventricular geometry on left atrium phasic function in obstructive sleep apnea syndrome: a multimodal echocardiography investigation
Source: BMC Cardiovasc Disord. 2021 Apr 24;21:209. doi: 10.1186/s12872-021-02018-1 (PMC8070276; doi:10.1186/s12872-021-02018-1)
Supplement: Supplementary file 1 — Additional file 1. Ethical approval. [file 12872_2021_2018_MOESM1_ESM.pdf]

山西医科大学第一医院伦理委员会快速审查批件

［2019］伦审字（K-ZK024）号

审查时间	2019-09-25	地点	办公楼五层会议室		
项目名称	阻塞性睡眠呼吸暂停高血压前期患者左室几何构型与炎症反应和氧化应激反应相关性的研究				
申办单位	山西医科大学第一医院	科室（专业）	超声影像科	临床研究负责单位	是
主要研究者	王健	职称	教授	联系电话	13834228878
审查文件	详见附件				
研究单位	山西医科大学第一医院				
出席委员	主审委员			委员	
	[signature]			[signature]	
审查意见	1.送审材料情况：符合伦理审查要求，研究方案科学合理，对于受试者的保护措施得当，主要研究者参加过 GCP 培训，具有开展临床试验的经验和能力。<br>2.临床试验过程中注意事项：注意对受试者的保护，如果发生病情加剧或严重不良事件，应及时报告伦理委员会。<br>3.试验要求：严格执行方案，遵守 GCP 规定，研究启动后每年将研究进展情况向伦理委员会通报，研究结束时需向伦理委员会提交备案。<br>4.内容修改要求：如临床试验方案、知情同意书的任何修改，主要研究单位更换，应及时通知伦理委员会，重新审查，获得批准后执行。<br>5. 该批件有效期为 3 年，本伦理委员会需要对该项目进行年度跟踪审查（1 年内未完成者）；在有效期内未完成者，请在有效期前 1 个月内向伦理委员会提交延期申请。<br>6.该批件为快速审查批件，如最近一次会议审查无异议，此批件效力等同正式批件；如有不同意见，即时作废，以会议审查结论为准。					
审查结论	[signature]	主任签字、盖章	[signature]	日期	2019 年 9 月 27 日

声明：本伦理委员会的组成及工作程序符合国家食品药品监督管理总局颁布的“药物临床试验质量管理规范”中的相关要求及其所遵循的 ICH-GCP 指导原则。

附件：

阻塞性睡眠呼吸暂停高血压前期患者左室几何构型与炎症反应和氧化应激反应相关性的研究

审阅材料一览表

序号	材料名称	版本号	日期
1	伦理审查申请表		
2	知情同意书		
3	研究方案		
