# Supplementary material for: The impact of left ventricular geometry on left atrium phasic function in obstructive sleep apnea syndrome: a multimodal echocardiography investigation
Source: BMC Cardiovasc Disord. 2021 Apr 24;21:209. doi: 10.1186/s12872-021-02018-1 (PMC8070276; doi:10.1186/s12872-021-02018-1)
Supplement: Supplementary file 2 — Additional file 2. Certificate of english editing. [file 12872_2021_2018_MOESM2_ESM.pdf]

CERTIFICATE OF ENGLISH EDITING

This document certifies that the paper listed below has been edited to ensure that the language is clear and free of errors. The edit was performed by professional editors at Editage, a division of Cactus Communications.The intent of the author's message was not altered in any way during the editing process. The quality of the edit has been guaranteed, with the assumption that our suggested changes have been accepted and have not been further altered without the knowledge of our editors.

Title Of The Paper

The impact of left ventricular geometry on left atrium phasic function in obstructive sleep apnea syndrome: A multimodal echocardiography investigation

Authors

Yong Zhang et al

Job Code

IYBIO_603

Signature

Vikas Narang,
Chief Operating Officer,
Editage

Date of Issue
March 27, 2021

Editage, a brand of Cactus Communications, offers professional English language editing and publication support services to authors engaged in over 500 areas of research. Through its community of experienced editors, which includes doctors, engineers, published scientists, and researchers with peer review experience, Editage has successfully helped authors get published in internationally reputed journals. Authors who work with Editage are guaranteed excellent language quality and timely delivery.

Contact Editage

Worldwide	Japan	Korea	China	Brazil
request@editage.com	submissions@editage.com	submit-korea@editage.com	fabiao@editage.cn	contato@editage.com
+1(833)979-0061	+81 0120-50-2987	02-3478-4396	400-005-6055	+5508000474773
www.editage.com	www.editage.jp	www.editage.co.kr	www.editage.cn	www.editage.com.br
